# Ecological Stress Tolerance and Biofilm-Associated Persistence in Opportunistic Bacteria Isolated from Livestock Faeces

**DOI:** 10.3390/pathogens15090935

**Published:** 2026-09-04

**Authors:** Catarina Silva, Telma de Sousa, João Rodrigues, Gilberto Igrejas, Patrícia Poeta

**Affiliations:** 1 Microbiology and Antibiotic Resistance Team (MicroART), Department of Veterinary Sciences, University of Trás-os-Montes and Alto Douro (UTAD), 5000-801 Vila Real, Portugal; xusilva2002@gmail.com (C.S.); telmaslsousa@hotmail.com (T.d.S.); 2 Functional Genomics and Proteomics Unit, University of Trás-os-Montes and Alto Douro (UTAD), 5000-801 Vila Real, Portugal; gigrejas@utad.pt; 3 Associated Laboratory for Green Chemistry (LAQV-REQUIMTE), Faculty of Sciences and Technology, Universidade NOVA de Lisboa, 2829-516 Caparica, Portugal; 4 Infectious Diseases Department, National Institute of Health Dr. Ricardo Jorge (INSA), 1649-016 Lisbon, Portugal; joao.rodrigues@insa.min-saude.pt; 5 Department of Genetics and Biotechnology, University of Trás-os-Montes and Alto Douro (UTAD), 5000-801 Vila Real, Portugal; 6 Veterinary and Animal Research Center (CECAV), University of Trás-os-Montes and Alto Douro (UTAD), 5000-801 Vila Real, Portugal; 7 Associate Laboratory for Animal and Veterinary Sciences (AL4AnimalS), University of Lisbon, 1300-477 Lisboa, Portugal

**Keywords:** livestock, one health, persistence, environmental stresses, *Pseudomonas putida*, *Pseudomonas fulva*, *Alcaligenes faecalis*

## Abstract

Livestock faeces constitute an important environmental interface within the One Health framework, as bacteria shed by animals are directly exposed to external environmental conditions, where they may persist and subsequently disseminate across the human, animal, and environmental sectors. The ability of these microorganisms to tolerate environmental stresses is therefore critical for their persistence and ecological distribution. In this study, the responses of 5 *Pseudomonas putida*, 15 *Pseudomonas fulva*, and 12 *Alcaligenes faecalis* isolates recovered from livestock faeces to different environmental stress conditions were investigated. Bacterial growth was evaluated under different temperatures, increasing NaCl concentrations, and carbon starvation, while biofilm formation was assessed using the microtiter plate assay. Growth was markedly affected by all stress conditions, although substantial variability was observed among isolates of the same species. Despite reduced growth under the most adverse conditions, all three species remained capable of growing across a wide range of environmental stresses. *A. faecalis* exhibited greater salt tolerance and biofilm-forming capacity than *Pseudomonas* spp., with biofilm formation detected in almost all isolates of both species. Furthermore, no statistically significant correlations were detected among the evaluated stress tolerance traits. These findings demonstrate that livestock-associated *P. putida*, *P. fulva*, and *A. faecalis* possess physiological characteristics that promote persistence under diverse environmental conditions, highlighting the potential role of livestock faeces as environmental reservoirs of opportunistic bacteria within the One Health context.

## 1. Introduction

The One Health concept recognizes that the health of humans, animals and the environment is intrinsically interconnected [1]. Environmental reservoirs play a central role in this continuum by acting as interfaces where microorganisms originating from humans and animals can persist, interact with resident microbial communities and potentially re-enter new hosts [2]. Understanding the factors that influence bacterial survival outside the host is therefore essential for characterizing their environmental fate and potential impact across the One Health interface.

The continuous expansion of livestock production has been accompanied by a substantial increase in the amount of animal manure generated worldwide [3,4], reinforcing the close interconnection between all sectors. Animal faeces represent a major vehicle for the release of intestinal microorganisms into agricultural soils, manure-amended fields and surrounding water systems, where they may persist, adapt and interact with resident microbial communities [5].

Most studies on livestock-associated microorganisms have focused on classical zoonotic pathogens and antimicrobial-resistant bacteria [6,7]. Nevertheless, livestock faeces also release a wide range of opportunistic bacteria into agricultural ecosystems [5]. Although many of these bacteria are frequently detected in both animal-associated and environmental niches [8], little is known about the persistence-associated traits that enable their survival following excretion.

Bacteria are among the most successful forms of life on Earth, being endowed with diverse mechanisms to survive in a variety of habitats, including inhospitable environments with extreme and changing conditions [9].

Specifically, species belonging to the genus *Pseudomonas* are ubiquitous inhabitants of soil, water and the rhizosphere and are recognized for their exceptional metabolic versatility, enabling survival under highly variable environmental conditions [10]. Similarly, *Alcaligenes faecalis*, traditionally regarded as an environmental microorganism, has been increasingly recovered from soil, water, animals and clinical settings [11]. These bacteria exhibit phenotypic characteristics that favour survival across multiple ecological compartments, facilitating persistence outside the host and increasing opportunities for environmental dissemination.

This is of particular concern because although commonly found in environmental habitats, *P. putida*, *P. fulva*, and *A. faecalis* can act as opportunistic pathogens, causing disease when favourable conditions or host susceptibility allow infection. Clinical infections have been reported for all three species, including cellulitis with bacteraemia caused by *P. putida* [12], septic shock associated with *P. fulva* [13], and contact lens-related keratitis and respiratory infection in rabbits caused by *A. faecalis* [14,15]. Their ability to persist under diverse environmental conditions, together with their documented opportunistic pathogenicity, makes these bacteria particularly relevant for investigating bacterial persistence and stress tolerance within a One Health framework.

The ability to remain viable under adverse conditions, commonly referred to as environmental persistence, represents a key component of bacterial ecological fitness. This capacity allows microorganisms to survive outside the host while maintaining their circulation across different environmental compartments [16].

Exogenous stresses, such as changes in temperature, salinity, and nutrient starvation, trigger bacterial stress response mechanisms that favour survival under adverse conditions. These responses promote persistence and adaptive evolution, allowing bacteria to withstand environmental challenges over extended periods. During persistence, bacteria maintain active survival mechanisms that can facilitate the accumulation of heritable genetic changes, either through increased mutagenesis or horizontal gene transfer. Consequently, persistent bacterial populations may serve as a reservoir for the emergence and dissemination of antimicrobial resistance [16].

In parallel, persistent bacterial populations embedded within biofilms, remain in close physical proximity for extended periods, creating favourable conditions for horizontal gene transfer through conjugation, transformation and transduction [16,17].

Although previous studies have investigated individual aspects of bacterial persistence in livestock-associated environments, little is known about how different persistence-associated phenotypes vary among livestock-associated isolates and whether these traits are related to each other following faecal excretion. To address this knowledge gap, we phenotypically characterized persistence-associated traits of opportunistic bacteria recovered from livestock faeces by evaluating their ability to withstand nutrient deprivation, osmotic and temperature stresses, together with their capacity to form biofilms. In addition, correlation analyses were performed to determine whether the evaluated stress-tolerance and persistence-associated phenotypes were significantly associated. By integrating these phenotypic characteristics, this work provides insight into the adaptive potential of livestock-associated opportunistic bacteria and their possible role as persistent environmental microorganisms within agricultural ecosystems.

## 2. Materials and Methods

### 2.1. Isolation and Identification of Bacterial Isolates

A total of 32 bovine and ovine faecal samples were collected during the winter season, including 3 bovine and 29 ovine samples. Each faecal sample was first inoculated into Brain Heart Infusion broth (BHI; Liofilchem, Roseto degli Abruzzi, Italy) and incubated for 24 h at 37 °C. Subsequently, 10 uL of the enriched cultures were streaked onto BHI agar (Liofilchem, Roseto degli Abruzzi, Italy) for 24 h, 37 °C. Following incubation, one well-isolated colony representative of the predominant colony morphology observed for each sample was selected for purification and subsequent identification. The identification was performed by matrix-assisted laser desorption/ionization time-of-flight mass spectrometry (MALDI-TOF MS), using the VITEK^®^ MS system, according to the manufacturer’s instructions (bioMérieux, Lisbon, Portugal).

### 2.2. Temperature Tolerance Assay

The effect of temperature on bacterial growth was evaluated using a broth microdilution assay based on the method described by Wang et al. [18]. Briefly, a single colony of each isolate was inoculated into Tryptic Soy Broth (TSB; Liofilchem, Roseto degli Abruzzi, Italy) and incubated at 37 °C for 24 h with shaking (150 rpm). Overnight cultures were diluted in fresh TSB to a turbidity equivalent to 0.5 McFarland. Subsequently, 200 μL of each standardized bacterial suspension was dispensed into sterile 96-well microtiter plates. Plates were incubated for 24 h at 6, 25, 37, and 42 °C. Bacterial growth was determined by measuring the optical density at 630 nm (OD630) using a microplate reader (EZ Read 800 Plus, Biochrom, Cambridge, UK). Wells containing sterile TSB served as blanks, and blank-corrected OD values were used for data analysis. All isolates were made in triplicate. Optical density values were corrected by subtracting the corresponding blank value.

### 2.3. Salinity Tolerance Assay

The effect of salinity on bacterial growth was evaluated using a modified broth microdilution assay based on the method described by Worrich et al. [19]. Briefly, a single colony of each isolate was inoculated into TSB and incubated at 37 °C for 24 h with shaking (150 rpm). Overnight cultures were diluted in fresh TSB to a turbidity equivalent to 0.5 McFarland.

Aliquots of 20 μL of each standardized bacterial suspension were dispensed into sterile 96-well microtiter plates containing 180 μL of TSB supplemented with NaCl to final concentrations of 0, 1, 2, 4, 6, or 8% (*w*/*v*). Each isolate was tested in triplicate at each NaCl concentration. Plates were incubated at 37 °C for 24 h with shaking (150 rpm).

Bacterial growth was determined by measuring the optical density at 630 nm (OD630) using a microplate reader (EZ Read 800 Plus, Biochrom). Wells containing sterile TSB supplemented with the corresponding NaCl concentration served as blank, and blank-corrected optical density values were calculated.

The mean and standard deviation (SD) of the three replicates were calculated to assess variability among isolates. The corresponding values are presented in Supplementary Materials Tables S6–S11.

To compare growth across salinity conditions, relative growth (%) was calculated using the blank-corrected OD values according to the following equation:

$$Relative\;growth\;\left(\%\right)=\frac{ODcorreted,\;NaCl}{ODcorrected,\;control}\times 100$$

where *ODcorrected*, *NaCl* is the blank-corrected optical density of an isolate at a given NaCl concentration, and *ODcorrected*, control is the blank-corrected optical density of the same isolate grown in TSB without NaCl, which was considered to represent 100% growth [20]. This normalization allowed the relative growth of each isolate at each NaCl concentration to be expressed as a percentage of its growth under salt-free conditions.

### 2.4. Nutrient Starvation Assay

Bacterial survival under starvation conditions was evaluated using a modified carbon starvation assay based on the method described by Betts et al. [21]. Briefly, a single colony of each isolate was inoculated into TSB and incubated at 37 °C for 24 h with shaking (150 rpm). Subsequently, 5 μL of the overnight culture was transferred into 245 μL of fresh TSB and incubated under the same conditions until the cultures reached the exponential growth phase (OD630 = 0.5–0.6).

Cells were harvested by centrifugation (6000× *g*, 5 min, room temperature), and the pellets were washed twice with sterile phosphate-buffered saline (PBS; Sigma-Aldrich/Merck, Darmstadt, Germany; pH 7.2, 0.01 mol/L phosphate buffer, 0.137 mol/L NaCl) to remove residual nutrients. After the final wash, each pellet was resuspended in 1 mL of sterile M9 minimal salts medium (6.0 g/L Na_2_HPO_4_, 3.0 g/L KH_2_PO_4_, 0.5 g/L NaCl, and 1.0 g/L NH_4_Cl), with no exogenous carbon source added [22]. Subsequently, 500 μL of the suspension was transferred into 4.5 mL of fresh carbon-free M9 medium to initiate the starvation assay. An aliquot was immediately collected for viable cell enumeration (0 h), and the remaining cultures were incubated at 37 °C with shaking (150 rpm).

Viable cell counts were determined after 24, 48, and 72 h, and after 7 and 14 days, as well as after 4 and 6 weeks of incubation. At each sampling point, serial dilutions were prepared in sterile PBS, and 100 μL aliquots were spread onto Plate Count Agar (PCA; Liofilchem, Roseto degli Abruzzi, Italy). Plates were incubated at 37 °C for 24 h, after which CFU were enumerated. Viable cell concentrations were expressed as colony-forming units per millilitre (CFU/mL). Viable cell counts were used instead of optical density to assess bacterial survival during prolonged nutrient limitation, as CFU quantifies only viable cells capable of growth.

### 2.5. Biofilm Formation Assay

Biofilm formation was evaluated using a modified crystal violet microtiter plate assay based on a previously described method [23]. Briefly, a single colony of each isolate was inoculated into TSB and incubated at 37 °C for 24 h with shaking (150 rpm). Cultures were then diluted in fresh TSB to a turbidity equivalent to 0.5 McFarland.

Aliquots (150 μL) of each standardized bacterial suspension were dispensed into sterile 96-well polystyrene microtiter plates. Wells containing only TSB served as negative controls, while the biofilm-producing reference strain *Pseudomonas aeruginosa* ATCC^®^ 27853 was included as a positive control. Plates were incubated at 37 °C for 24 h with shaking (150 rpm).

Following incubation, the planktonic cultures were discarded, and the wells were gently washed twice with distilled water to remove non-adherent cells. 200 μL of pure methanol were added for 5–10 min to fix the biofilm. After this process, the methanol was removed and the film was allowed to dry. The attached biofilms were stained with 200 μL 0.1% (*w*/*v*) crystal violet for 10–15 min at room temperature. Excess stains were removed by washing the plates three to four times with distilled water, after which the plates were air-dried overnight. The bound crystal violet was solubilized with 200 μL 30% (*v*/*v*) acetic acid, and the absorbance was measured at 570 nm using a microplate reader (EZ Read 800 Plus, Biochrom).

Biofilm production was classified according to the criteria described by Stepanović et al. [23].

### 2.6. Statistical Analysis

To investigate potential associations among stress-related phenotypic traits, correlation analyses were performed using Spearman’s rank correlation coefficient (Spearman’s ρ). This non-parametric test was selected because it is more appropriate for biological data that does not necessarily follow a normal distribution or exhibit linear relationships. Unlike parametric correlation methods, Spearman’s test evaluates monotonic associations based on ranked values rather than absolute measurements, making it particularly robust for microbiological datasets.

The following correlations were investigated to determine whether different stress-adaptation mechanisms were associated: (i) biofilm formation versus bacterial growth at 42 °C, to evaluate whether thermotolerant isolates exhibited an enhanced capacity for biofilm formation; (ii) biofilm formation versus relative growth at 8% NaCl, to assess whether halotolerance was associated with increased biofilm production; (iii) biofilm formation versus bacterial survival following six weeks of nutrient starvation, to investigate whether long-term persistence under nutrient-limited conditions was linked to biofilm-forming ability; and (iv) bacterial growth at 42 °C versus relative growth at 8% NaCl, to determine whether thermotolerance and halotolerance represented related adaptive phenotypes.

To account for multiple correlation comparisons, *p*-values were adjusted using the Benjamini–Hochberg false discovery rate (FDR) correction. Statistical significance was considered at *p* < 0.05.

## 3. Results and Discussion

### 3.1. Bacterial Isolates and Species Identification

The isolates evaluated in the present study were recovered from bovine and ovine faecal samples using a selective culture medium. Species identification revealed five *P. putida*, fifteen *P. fulva*, and twelve *A. faecalis* isolates (Table S1, Supplementary Materials). Their ability to grow under selective conditions may reflect intrinsic physiological traits that enhance survival in the presence of inhibitory compounds. The subsequent identification of these isolates further highlights the diversity of environmental bacteria associated with livestock faeces.

Although these species are primarily regarded as environmental microorganisms, they have been repeatedly recovered from livestock-associated environments. *P. putida* and *P. fulva* are common inhabitants of soil, freshwater and the rhizosphere, but have also been isolated from animal faeces and agricultural environments, supporting their adaptation to the livestock–environment interface [24,25]. Likewise, *A. faecalis* is widely distributed in soil [26], water [27] and the intestinal tract of animals [11] and has frequently recovered from faecal material and manure, where it contributes to nutrient cycling and organic matter degradation [28]. These observations are consistent with the present findings and reinforce the view that livestock faeces constitute an important reservoir from which environmental bacteria can be released into surrounding ecosystems.

### 3.2. Effect of Temperature in Bacterial Growth

The selected temperatures were chosen to represent distinct environmental and physiological conditions. Incubation at 6 °C was used to assess bacterial survival under refrigeration conditions, which are relevant to environmental persistence and the conservation of animal-derived samples. Incubation at 25 °C represented typical environmental conditions, whereas 37 °C was selected as a host-associated physiological temperature. Finally, 42 °C represented an elevated-temperature condition, allowing the assessment of tolerance to thermal stress.

**Figure 1:**
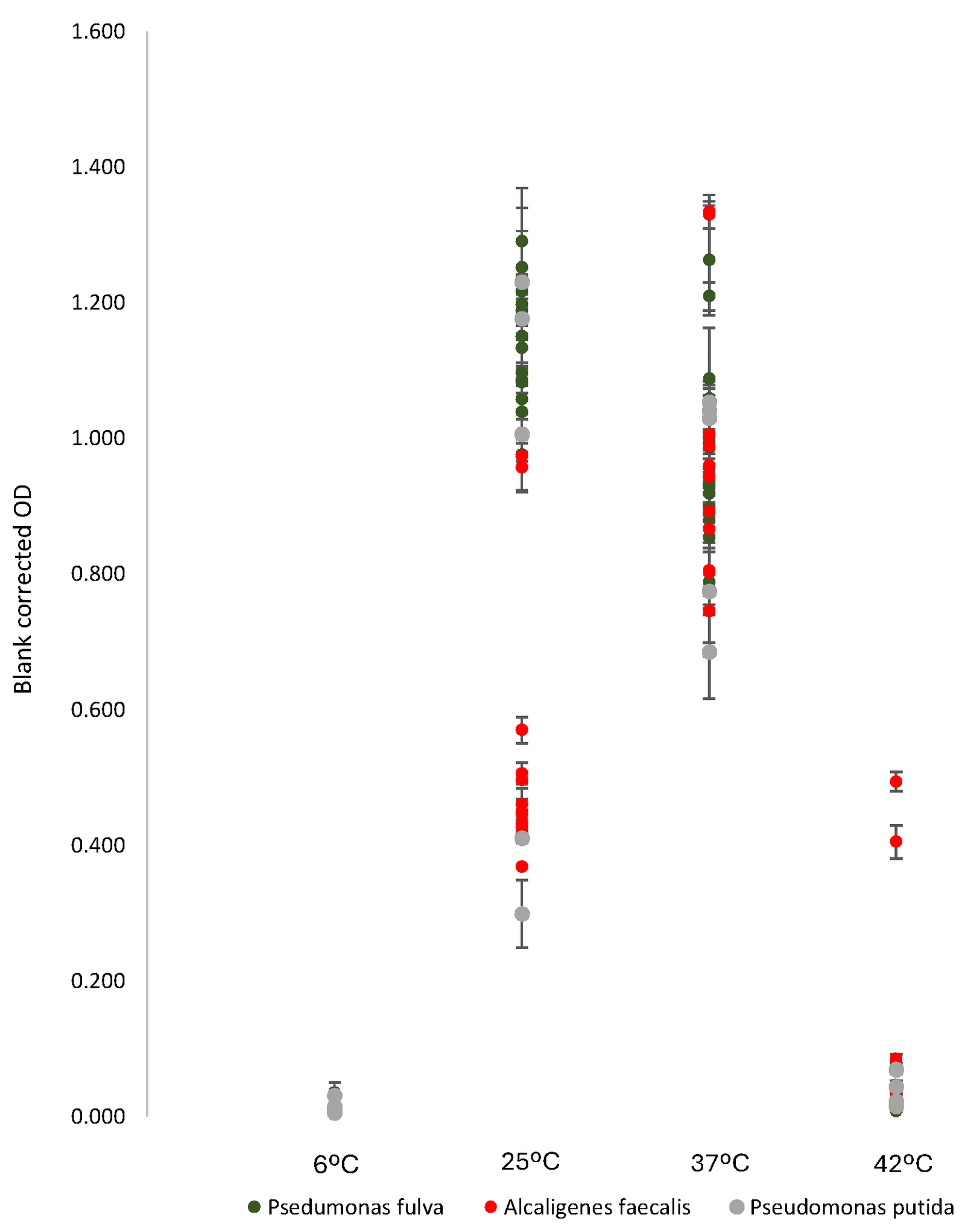
Effect of incubation temperature on the growth of the bacterial isolates. Each point represents an individual isolate. *Pseudomonas fulva* isolates are shown in dark green, *Pseudomonas putida* isolates are shown in grey, and *A. faecalis* isolates are shown in red. Vertical bars represent the standard deviation (SD) for each isolate.

These temperature conditions revealed clear differences in bacterial growth, with incubation temperature markedly influencing bacterial growth (Figure 1). At 6 °C, all isolates exhibited minimal growth, with blank-corrected OD values below 0.04. Growth increased substantially at 25 °C and remained high at 37 °C, although greater variability among isolates was observed at the latter temperature. In contrast, incubation at 42 °C resulted in a pronounced reduction in growth for most isolates.

The overall similarity in the phenotypic responses of *P. fulva* and *P. putida* across the experimental conditions evaluated justified discussing the two species together throughout this section. Species-specific observations are highlighted where relevant. Both species exhibited the highest overall growth at 25 °C, with most isolates reaching blank-corrected OD values above 1.0. Growth remained high at 37 °C despite increased inter-isolate variability, whereas a marked decline was observed at 42 °C, contrasting with the well-documented ability of *Pseudomonas aeruginosa* to grow efficiently at this temperature [29].

*A. faecalis* isolates showed OD values numerically lower than those observed for both *Pseudomonas* spp. at 25 °C, with OD values ranging from 0.37 to 0.57, but showed improved growth at 37 °C, with most isolates reaching OD values close to or exceeding 0.9. At 42 °C, growth decreased markedly. However, *A. faecalis* isolates 31 and 32 maintained relatively high OD values compared with the remaining isolates, indicating greater tolerance to elevated temperature.

In the present study, bacterial growth was strongly influenced by incubation temperature (Tables S2–S5, Supplementary Materials), reflecting the environmental conditions encountered in natural habitats such as soil, freshwater and the rhizosphere, where bacteria are exposed to seasonal and spatial temperature fluctuations [30]. These fluctuations may be further intensified by livestock manure storage practices [31] and climate change [32], significantly affecting bacterial growth, survival and environmental persistence.

Given the limited information available on the responses of *P. fulva* to environmental stress, comparisons throughout the discussion are based primarily on studies of *P. putida* and the genus *Pseudomonas*, which provide the best-characterized models of environmental adaptation.

Growth was minimal at 6 °C for all isolates, indicating very limited measurable biomass production under this condition. However, low optical density does not necessarily indicate loss of viability, as bacteria may maintain metabolic activity and cellular viability under conditions in which growth is strongly reduced. Previous studies have shown that *P. putida* can adapt to low-temperature environments through extensive metabolic and physiological adjustments, including modifications in amino acid uptake and metabolism, membrane composition, energy metabolism and respiratory pathways. These adaptations preserve cellular homeostasis and allow bacterial survival and persistence despite substantially reduced growth rates [33].

The highest growth was observed between 25 and 37 °C, consistent with the mesophilic nature of both *Pseudomonas* spp. and *A. faecalis*. *Pseudomonas* isolates generally exhibited greater growth at 25 °C than *A. faecalis*, suggesting better adaptation to moderate environmental temperatures, which agrees with the widespread occurrence of environmental *Pseudomonas* species in soil and aquatic ecosystems. In contrast, *A. faecalis* showed improved growth at 37 °C, indicating a preference for slightly warmer conditions, as previously reported for this species [34].

Growth declined markedly at 42 °C for most isolates, reflecting the physiological constraints imposed by temperatures approaching the upper growth limit of mesophilic bacteria. Similar observations have been reported for *P. putida*, whose critical growth temperature has been described to be approximately 40 °C, with little or no growth occurring above this threshold [35]. Nevertheless, two *A. faecalis* isolates (31 and 32) maintained comparatively higher growth at 42 °C than the remaining isolates, indicating that temperature tolerance may vary among isolates of the same species.

In natural ecosystems, bacteria are continuously exposed to temperature fluctuations, which are expected to become more frequent and intense under current climate change scenarios. Such environmental changes may influence the persistence and dispersal of ubiquitous environmental microorganisms by altering the ecological conditions in which they survive. Therefore, bacterial populations capable of tolerating a broad temperature range may have a greater capacity to persist in environmental reservoirs and spread across interconnected environmental, animal and human compartments, reinforcing the ecological relevance of these adaptive traits within a One Health perspective [36].

### 3.3. Effect of Salinity on Bacterial Growth

**Figure 2:**
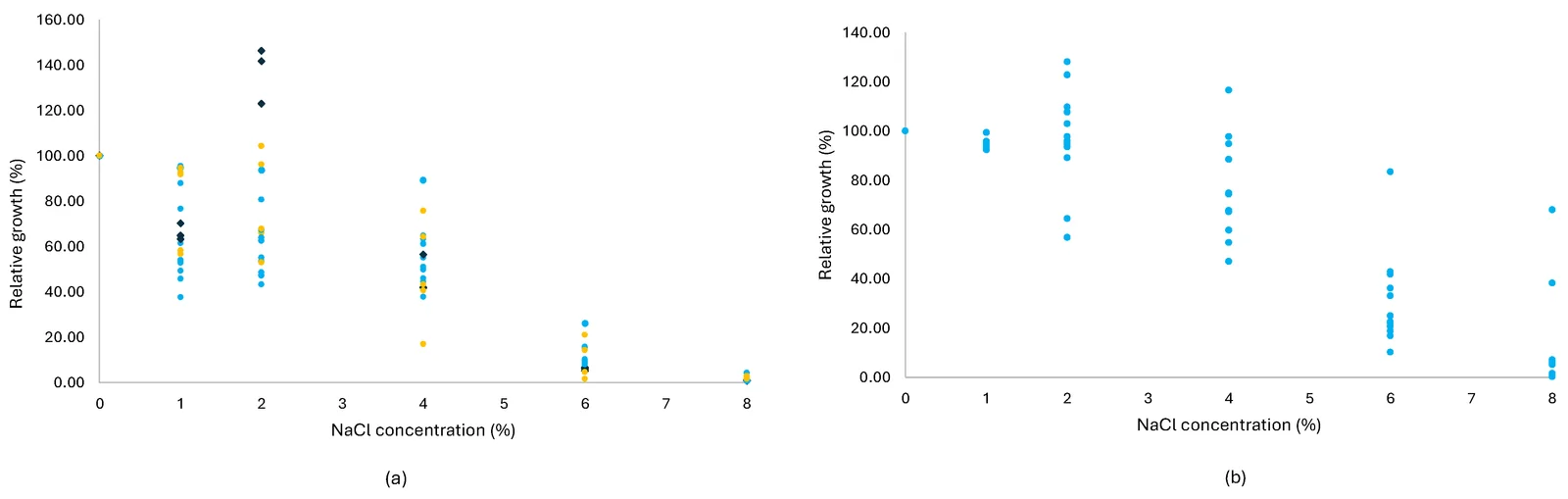
Relative growth (%) of *Pseudomonas* spp. (**a**) and *A. faecalis* (**b**) isolates at increasing NaCl concentrations. In panel (**a**), *P. putida* isolates are shown in yellow with circular markers, while *P. fulva* isolates are shown in blue with circular markers. *P. fulva* isolates 12, 13, and 14 are highlighted in dark blue with diamond-shaped markers. Each point represents an individual isolate.

Salinity influenced bacterial growth (Figure 2), although all isolates remained capable of growth across the range of NaCl concentrations tested (Tables S6–S11, Supplementary Materials).

Most *Pseudomonas* spp. isolates showed a slight reduction in relative growth at 1% NaCl compared with the control, followed by a recovery or increase at 2% NaCl. Notably, *P. fulva* isolates 12, 13, and 14 exhibited enhanced growth at intermediate salinity, with relative growth exceeding 120% at 2% NaCl, indicating that these isolates produced more than 20% greater endpoint biomass than under the no-salt control condition. Most isolates maintained more than 50% relative growth at 4% NaCl, demonstrating considerable tolerance to intermediate NaCl concentrations within the range tested. Although growth was reduced at higher salt concentrations, several isolates remained viable at 6% NaCl, and residual growth was still detected at 8% NaCl.

*A. faecalis* isolates displayed the highest overall tolerance to salinity. Relative growth remained close to or above control in most isolates at 1% and 2% NaCl, and several strains maintained substantial growth at elevated salt concentrations. Isolate 25 showed exceptional halotolerance, exhibiting relative growth above 100% at both 2% and 4% NaCl and retaining 83.5% and 68.0% of its control growth at 6% and 8% NaCl, respectively. Isolate 23 also demonstrated remarkable tolerance, maintaining 42.8% and 38.2% relative growth at 6% and 8% NaCl, respectively, while isolates 31 and 32 retained more than 35% relative growth at 6% NaCl.

Overall, the results demonstrate that both genera were capable of growth under elevated salinity, although the degree of halotolerance varied among isolates. *A. faecalis* exhibited the highest salt tolerance, with several isolates maintaining substantial growth even at 6–8% NaCl, whereas *Pseudomonas* spp. generally tolerated salinities up to 4% NaCl and a smaller number of isolates remained capable of appreciable growth at higher salt concentrations. Considerable variability in salt tolerance was also observed among isolates belonging to the same species, highlighting substantial intraspecific variation in the response to saline stress.

To investigate these differences, NaCl concentrations ranging from 0 to 8% were selected to generate a gradient of osmotic stress encompassing conditions representative of non-saline and moderately saline environments, as well as more extreme saline conditions. Such a gradient reflects the wide range of salinities that bacteria may encounter in soils and aquatic environments, including those affected by irrigation practices, salinization processes and other environmental factors.

Osmotic stress is one of the major environmental constraints that soil bacteria encounter during growth and survival [37].

Environmental *Pseudomonas* species are generally regarded as moderately halotolerant rather than truly halophilic [38], and previous studies have demonstrated gradual reductions in growth with increasing salinity [39]. The response observed in the present study is therefore consistent with the known physiology of the genus.

Interestingly, several *Pseudomonas* isolates exhibited lower growth at 1% NaCl than under control conditions but showed improved growth at 2% NaCl, with some isolates even exceeding the growth observed in the absence of added NaCl. This response contrasts with most previous studies, which generally report a progressive decline in *Pseudomonas* growth as NaCl concentration increases [37]. This observation may reflect strain-specific differences in osmoadaptation. *Pseudomonas* spp. are known to tolerate moderate osmotic stress through the synthesis or uptake of compatible solutes, including glycine betaine, glutamate and trehalose, which preserve cellular turgor and enzyme function under saline conditions. These mechanisms are accompanied by the regulation of genes involved in osmoprotectant transport and biosynthesis, enabling cells to maintain metabolic activity and growth under moderate osmotic stress [40].

However, as salinity increases, the energetic cost associated with osmoadaptation becomes progressively greater, leading to reduced growth, particularly at NaCl concentrations above 3–4% [39].

*A. faecalis* generally showed higher OD values than *Pseudomonas* spp. at elevated NaCl concentrations, suggesting a greater capacity for growth under these conditions. Several isolates maintained growth close to or above the control at 2% NaCl, while isolate 25 retained nearly 70% of its initial growth even at 8% NaCl. These findings suggest that some *A. faecalis* isolates possess efficient osmo-adaptive mechanisms, enabling growth under relatively high osmotic stress. This observation is consistent with previous reports describing members of this genus as halotolerant bacteria capable of maintaining growth under moderate to high salinity [41]. The greater salt tolerance observed among *A. faecalis* isolates may contribute to their ability to remain metabolically active under a broader range of osmotic conditions, potentially favoring their persistence and subsequent dissemination across livestock-associated environmental compartments. Nevertheless, substantial variability among isolates indicates that salinity tolerance is strongly strain dependent.

### 3.4. Bacterial Survival Under Nutrient Starvation

Both *Pseudomonas* spp. and *A. faecalis* exhibited similar CFU values throughout the entire carbon starvation incubation period, with little effect on bacterial viability during the first two weeks and a gradual decline only after four weeks. At time zero, viable cell concentrations ranged from 7.03 to >8.78 log_10_ (CFU/mL). After 24 h, viable cell concentrations increased to approximately 10^8^ CFU/mL, with almost all isolates exceeding the upper counting limit of the assay. This pattern persisted throughout the 14-day incubation period, indicating that these bacteria can maintain high levels of culturability and persist under nutrient-limited conditions. Only isolates 16 and 37 produced countable plates after 24 h, with viable counts of 8.15 and 8.24 log_10_ (CFU/mL), respectively, before also exceeding the upper counting limit of the assay at subsequent sampling points.

Viable counts became quantifiable again only after four weeks of incubation, as bacterial populations declined below the upper counting limit of the assay. Nevertheless, viable concentrations remained high, ranging from 7.79 to 8.68 log_10_ (CFU/mL), indicating prolonged persistence under carbon-limited conditions. By six weeks, a further modest decline in viability was observed, with viable counts ranging from 7.26 to 7.66 log_10_ (CFU/mL), indicating that prolonged carbon starvation eventually affected bacterial survival, although all isolates remained culturable throughout the experiment, with quantifiable populations remaining above 10^7^ CFU/mL throughout the experiment (Table S12, Supplementary Materials).

Changes in colony morphology were qualitatively observed throughout the carbon starvation assay. As the incubation period progressed, colonies of several isolates exhibited alterations in morphology, including differences in colony size and appearance compared with those observed at the beginning of the experiment. In some cases, morphological variability was also observed among colonies derived from the same isolate at a given sampling time, resulting in the coexistence of distinct colony morphotypes.

The absence of an evident reduction in culturable counts during the first 14 days suggests that these environmental isolates possess effective survival mechanisms that enable persistence under nutrient-limited conditions. However, because several measurements exceeded the upper counting limit of the assay, the magnitude of changes during this period could not be quantitatively determined.

The decline in viable cell counts observed after four weeks suggests that these adaptive mechanisms gradually become less effective during prolonged starvation. As intracellular energy reserves are progressively depleted and cellular maintenance can no longer be fully sustained, bacterial viability eventually begins to decrease despite the activation of long-term survival responses.

Long-term survival during carbon starvation has been extensively described for environmental bacteria, particularly members of the genus *Pseudomonas* [42].

Under nutrient limitation, bacterial cells undergo extensive physiological and metabolic adjustments, including reduced metabolic activity, activation of stress-response pathways, and increased resource conservation, allowing prolonged survival in the absence of active growth [43].

In consequence to carbon starvation, cells develop phenotypic heterogeneity. The progressive changes in colony size observed during starvation, together with the coexistence of large and small colonies on the same plates, are consistent with the development of phenotypic heterogeneity. Such heterogeneity is considered an adaptive strategy that enables bacterial populations to cope with fluctuating environmental conditions by generating subpopulations with distinct physiological states. Consequently, some cells resume growth more rapidly when transferred to nutrient-rich media, forming larger colonies, whereas others display delayed recovery and produce smaller colonies [42,44].

The sustained culturability observed in the present study indicates that the isolates were able to maintain viability under prolonged carbon limitation. This trait is particularly relevant following excretion, when bacteria transition from the nutrient-rich environment of the gastrointestinal tract to nutrient-poor environmental matrices such as soil and water. Such persistence may represent an important ecological advantage, allowing bacteria to survive prolonged periods of nutrient limitation, which are common in natural environments, until favourable growth conditions are restored.

### 3.5. Biofilm Formation

Biofilm formation was evaluated according to the following criteria: OD ≤ ODc (non-biofilm producer), ODc < OD ≤ 2 × ODc (weak biofilm producer), 2 × ODc < OD ≤ 4 × ODc (moderate biofilm producer), and OD > 4 × ODc (strong biofilm producer), where the optical density cut-off (ODc) was defined as: ODc = mean OD of the negative control + 3 × SD of the negative control [45].

Biofilm formation was evaluated for all *Pseudomonas* spp. and *A. faecalis* isolates (Table 1).

Among the 20 *Pseudomonas* isolates, 1 (5%) was classified as a strong biofilm producer, 2 (10%) as moderate, 15 (75%) as weak, and 2 (10%) as non-producers. In contrast, all 12 *A. faecalis* isolates exhibited biofilm formation, with 7 (58%) classified as strong and 5 (42%) as moderate biofilm producers.

Overall, the results demonstrate that *A. faecalis* showed higher biofilm formation values than *Pseudomonas* spp., although biofilm formation was consistently observed among almost all biofilm-producing isolates (Table S13, Supplementary Materials).

Biofilm formation is widely recognised as an important bacterial survival strategy, facilitating persistence on abiotic surfaces while increasing tolerance to environmental stresses [9]. In the present study, biofilm formation was detected in both bacterial groups.

Among *Pseudomonas* isolates, 90% produced detectable biofilms, whereas only 2 isolates were classified as non-producers under the experimental conditions. Members of the genus *Pseudomonas* are generally considered efficient biofilm formers. However, considerable strain-to-strain variability has been widely reported. Environmental *Pseudomonas* isolates have been described as exhibiting varying levels of biofilm formation when evaluated using crystal violet assays [46,47], demonstrating that biofilm formation depends strongly on the genetic background of individual isolates [48] as well as on environmental conditions [9]. In the present study, although most *Pseudomonas* isolates were classified as weak biofilm producers, moderate and strong biofilm-producing isolates were also identified, further highlighting the variability in biofilm-forming capacity among isolates. This variability does not necessarily indicate an intrinsic inability to form biofilms but may instead reflect strain-specific genetic differences and the influence of the experimental conditions, particularly given that these environmental isolates were evaluated outside their natural habitats.

All *A. faecalis* isolates produced detectable biofilms, indicating a consistent and relatively high capacity to adhere to abiotic surfaces and form substantial biofilm biomass under the experimental conditions. Quantitative studies on biofilm formation by *A. faecalis* are relatively scarce. However, previous reports have demonstrated that this species can produce an extracellular polymeric matrix and establish biofilms, supporting its capacity for biofilm formation observed in the present study [49].

Overall, biofilm formation was observed across the isolates, although some degree of intra- and interspecies variability was evident. These findings suggest that biofilm formation may represent an important survival strategy for these bacteria, conferring ecological advantages by promoting surface colonisation and enhancing persistence under fluctuating environmental conditions.

### 3.6. Statistical Analysis of Bacterial Responses

Correlation analyses revealed no statistically significant associations among the evaluated stress-related phenotypic traits (Table 2).

No statistically significant correlation was detected between bacterial growth at 42 °C and biofilm formation (Spearman’s ρ = 0.351, *p* = 0.042, FDR-adjusted *p* = 0.168). Similarly, no significant correlation was detected between biofilm formation and relative growth at 8% NaCl (Spearman’s ρ = 0.141, *p* = 0.428, FDR-adjusted *p* = 0.571). Survival after six weeks of nutrient starvation was also not significantly correlated with biofilm formation (Spearman’s ρ = −0.130, *p* = 0.463, FDR-adjusted *p* = 0.571). Finally, no significant correlation was observed between bacterial growth at 42 °C and relative growth at 8% NaCl (Spearman’s ρ = 0.078, *p* = 0.660, FDR-adjusted *p* = 0.660).

Overall, none of the evaluated stress-response traits showed a statistically significant association, indicating that the phenotypic responses varied independently across the studied isolates.

Although no significant associations were identified among the evaluated stress-response traits, these findings remain relevant from a One Health perspective. The ability of *P. fulva*, *P. putida* and *A. faecalis* to persist under different environmental stresses may contribute to their maintenance in environmental and animal-associated reservoirs, even in the absence of strong associations between individual phenotypic traits. Importantly, although these bacteria are generally considered of low risk to healthy individuals, they have been associated with severe infections in humans and animals. Therefore, characterizing their environmental persistence and stress tolerance is important for understanding their potential ecological and epidemiological relevance at an early stage, before their potential emergence as opportunistic pathogens. These findings contribute to a better understanding of bacterial persistence at the environment–animal–human interface and highlight the relevance of monitoring environmental bacteria within a One Health framework.

## 4. Conclusions

Taken together, the present results demonstrate that environmental isolates of *Pseudomonas* spp. and *A. faecalis* possess a broad capacity to withstand diverse environmental stressors. Although elevated temperature and salinity reduced bacterial growth in many isolates, viable growth was consistently maintained by at least a subset of isolates under all conditions tested. Furthermore, prolonged survival under nutrient limitation and the ability to form biofilms suggest that these bacteria possess multiple adaptive traits that enhance persistence in challenging environments. The considerable variability observed among isolates also highlights the importance of strain-specific physiological responses in determining environmental fitness. The absence of significant correlations among the evaluated stress-response traits further suggests that environmental persistence is not driven by a single adaptive phenotype but rather by multiple complementary physiological mechanisms that vary among isolates.

In natural ecosystems, bacteria associated with livestock are frequently exposed to fluctuating temperatures, osmotic stress, nutrient limitation and surface-associated lifestyles. The ability to tolerate these stressors is likely to promote long-term persistence following their release into the environment. Consequently, bacteria originating from livestock faeces may persist in soil and water and potentially spread through interconnected environmental compartments, including the food chain, thereby increasing opportunities for contact with animal and human hosts. Their resilience to multiple environmental stressors may also increase their capacity to withstand extreme climatic events, such as droughts, floods and heatwaves, which are expected to become more frequent under climate change. Such conditions may further facilitate bacterial persistence and spread to new geographic areas, thereby increasing the likelihood of transmission to animal and human hosts.

These findings reinforce the importance of considering environmental persistence within a One Health framework, where the environment acts as a critical interface linking animal and human populations.

From a practical perspective, these findings highlight the importance of considering livestock manure management as a potential factor influencing the environmental dissemination of bacteria capable of persisting under diverse environmental conditions. Manure treatment strategies such as composting may contribute to reducing bacterial persistence before manure is applied to agricultural land. In addition, appropriate storage conditions and defined storage periods, together with standardized protocols for manure handling, treatment and application, may be important to minimize the release and dissemination of viable bacteria into surrounding soil and water.

The present findings were obtained under controlled laboratory conditions and therefore represent the phenotypic potential of the studied isolates rather than direct evidence of persistence under field conditions. Future studies should therefore investigate the survival of these bacteria under in situ conditions, particularly during livestock manure storage, treatment and subsequent environmental exposure. In addition, genomic and multi-omics approaches could provide further insight into the molecular mechanisms underlying the observed stress-tolerance phenotypes and help identify determinants associated with environmental persistence. Such approaches would contribute to a more comprehensive understanding of bacterial adaptation outside the host and support the development of more effective strategies for monitoring and managing environmentally persistent bacteria within a One Health framework.

A better understanding of the adaptive mechanisms that enable bacterial survival outside the host will improve our ability to predict environmental persistence and assess the potential for dissemination of microorganisms of public health relevance.

## Figures and Tables

**Table 1:** Biofilm-forming ability of the bacterial. Mean OD values (±SD) were calculated from three independent replicates, and isolates were classified as non-, weak, moderate, or strong biofilm producers according to the established ODc criteria.

Species	*n*	Mean OD ± SD	Biofilm Classification
*Pseudomonas fulva*	15	0.055 ± 0.005	1/15 (7%) Moderate; 13/15 (86%) Weak; 1/15 (7%) Non-producer
		0.072 ± 0.017	
		0.071 ± 0.013	
		0.071 ± 0.009	
		0.069 ± 0.010	
		0.063 ± 0.006	
		0.071 ± 0.010	
		0.076 ± 0.009	
		0.152 ± 0.026	
		0.093 ± 0.012	
		0.091 ± 0.006	
		0.109 ± 0.025	
		0.058 ± 0.010	
		0.061 ± 0.007	
		0.071 ± 0.006	
*Pseudomonas putida*	5	0.103 ± 0.018	1/5 (20%) Strong; 1/5 (20%) Moderate; 2/5 (40%) Weak; 1/5 (20%) Non-producer
		0.099 ± 0.038	
		0.248 ± 0.049	
		0.138 ± 0.020	
		0.053 ± 0.003	
*Alcaligenes faecalis*	12	0.242 ± 0.027	7/12 (58%) Strong; 5/12 (42%) Moderate
		0.203 ± 0.037	
		0.196 ± 0.016	
		0.169 ± 0.028	
		0.223 ± 0.026	
		0.320 ± 0.053	
		0.169 ± 0.025	
		0.423 ± 0.045	
		0.239 ± 0.024	
		0.310 ± 0.045	
		0.260 ± 0.028	
		0.247 ± 0.033	
Positive Control		0.348 ± 0.058	Strong
Negative Control		0.049 ± 0.003	

**Table 2:** Spearman’s rank correlation analysis between biofilm formation and stress tolerance-related phenotypes.

Comparison	Spearman’s ρ	FDR-Adjusted *p*	Interpretation
Biofilm vs. Growth at 42 °C	0.351	0.168	No correlation observed
Biofilm vs. Relative growth at 8% NaCl	0.141	0.571	No correlation observed
Biofilm vs. Survival after 6 weeks of nutrient starvation	−0.130	0.571	No correlation observed
Growth at 42 °C vs. Relative growth at 8% NaCl	0.078	0.660	No correlation observed

FDR, False discovery rate.

## Data Availability

The data presented in this study are available from the corresponding author upon reasonable request.

## Supplementary Materials

The following supporting information can be downloaded at https://www.mdpi.com/article/10.3390/pathogens15090935/s1, Table S1: Identification of the bacterial isolates included in this study by MALDI-TOF mass spectrometry; Table S2: OD growth values of *A. faecalis*, *P. fulva*, and *P. putida* isolates following incubation at 6 °C; Table S3: OD growth values of *A. faecalis, P. fulva*, and *P. putida* isolates following incubation at 25 °C; Table S4: OD growth values of *A. faecalis*, *P. fulva* and *P. putida* isolates following incubation at 37 °C. NC; Table S5: OD growth values of *A. faecalis*, *P. fulva*, and *P. putida* isolates following incubation at 42 °C; Table S6: Corrected OD values, standard deviations (SD), and relative growth (%) of *A. faecalis*, *P. fulva*, and *P. putida* isolates grown in NaCl-free medium (0% NaCl); Table S7: Corrected OD values, SD and relative growth (%) of *A. faecalis*, *P. fulva*, and *P. putida* isolates grown in medium supplemented with 1% NaCl; Table S8: Corrected OD values, SD, and relative growth (%) of *A. faecalis*, *P. fulva*, and *P. putida* isolates grown in medium supplemented with 2% NaCl; Table S9: Corrected OD values, SD, and relative growth (%) of *A. faecalis*, *P. fulva* and *P. putida* isolates grown in medium supplemented with 4% NaCl; Table S10: Corrected OD values, SD, and relative growth (%) of *A. faecalis*, *P. fulva* and *P. putida* isolates grown in medium supplemented with 6% NaCl; Table S11: Corrected OD values, SD, and relative growth (%) of *A. faecalis*, *P. fulva* and *P. putida* isolates grown in medium supplemented with 8% NaCl; Table S12: Viable bacterial counts (log_10_ CFU/mL) of *P. fulva*, *P. putida*, and *A. faecalis* isolates at each sampling time during the nutrient starvation assay; Table S13: OD values obtained for each isolate in eight replicates, together with the mean OD, and the corresponding standard deviation, used for biofilm formation analysis.

## Abbreviations

The following abbreviations are used in this manuscript:

BHI	Brain Heart Infusion
CFU	Colony-forming units
FDR	False discovery rate
KH_2_PO_4_,	Potassium dihydrogen phosphate
MALDI-TOF MS	Matrix-Assisted Laser Desorption/Ionization Time-of-Flight Mass Spectrometry
mL	Millilitre
NaCl	Sodium chloride
Na_2_HPO_4_,	Disodium hydrogen phosphate
NC	Negative control
NH_4_Cl	Ammonium chloride
nm	Nanometre
OD	Optical density
ODc	Optical density cut-off
PBS	Phosphate-buffered saline
PC	Positive control
PCA	Plate Count Agar
Rep	Replicate
rpm	Revolutions per minute
SD	Standard deviation
TNTC	Too numerous to count
TSB	Tryptic Soy Broth
μL	Microlitre
