# Supplementary material for: Ecological Stress Tolerance and Biofilm-Associated Persistence in Opportunistic Bacteria Isolated from Livestock Faeces
Source: Pathogens. 2026 Sep 4;15(9):935. doi: 10.3390/pathogens15090935 (PMC13610434; doi:10.3390/pathogens15090935)
Supplement: Supplementary file 1 [file pathogens-15-00935-s001.zip › pathogens-4502429-supplementary.pdf]

Supplementary Materials

Table S1 Identification of the bacterial isolates included in this study by MALDI-TOF mass spectrometry.

Isolate	Identification	Score
	**Ovine**	
1	*Pseudomonas putida*	9.159
2	*Pseudomonas fulva*	9.557
3	*Pseudomonas fulva*	9.236
4	*Pseudomonas fulva*	9.064
5	*Pseudomonas fulva*	9.159
6	*Pseudomonas fulva*	9.539
7	*Pseudomonas fulva*	6.378
9	*Pseudomonas fulva*	9.541
10	*Pseudomonas fulva*	9.587
11	*Alcaligenes faecalis*	9.583
12	*Pseudomonas fulva*	9.319
13	*Pseudomonas fulva*	9.330
14	*Pseudomonas fulva*	9.205
15	*Pseudomonas fulva*	6.618
16	*Pseudomonas fulva*	9.212
17	*Pseudomonas putida*	6.676
19	*Alcaligenes faecalis*	9.701
20	*Alcaligenes faecalis*	9.746
23	*Alcaligenes faecalis*	9.640
25	*Alcaligenes faecalis*	9.662
26	*Alcaligenes faecalis*	9.435
27	*Pseudomonas fulva*	9.208
28	*Alcaligenes faecalis*	9.754
29	*Alcaligenes faecalis*	9.662
30	*Alcaligenes faecalis*	9.601
31	*Alcaligenes faecalis*	9.499
32	*Alcaligenes faecalis*	9.624
33	*Pseudomonas fulva*	9.205
34	*Alcaligenes faecalis*	9.717
	**Bovine**	
37	*Pseudomonas putida*	9.523
38	*Pseudomonas putida*	7.111
42	*Pseudomonas putida*	8.283

Table S2 OD growth values of *A. faecalis, P. fulva,* and *P. putida* isolates following incubation at 6°C. NC: Negative Control; PC: Positive Control.

				6ºC		
**Isolate**	**Rep 1**	**Rep 2**	**Rep 3**	**Mean OD**	**OD corrected**	**Standard deviation**
1	0.071	0.076	0.077	0.075	0.015	0.0026
2	0.069	0.072	0.072	0.071	0.011	0.0014
3	0.057	0.079	0.084	0.073	0.014	0.0117
4	0.066	0.065	0.068	0.066	0.007	0.0012
5	0.066	0.064	0.07	0.067	0.007	0.0025
6	0.065	0.066	0.066	0.066	0.006	0.0005
7	0.074	0.079	0.078	0.077	0.017	0.0022
9	0.067	0.068	0.069	0.068	0.008	0.0008
10	0.115	0.079	0.092	0.095	0.036	0.0149
11	0.071	0.076	0.076	0.074	0.015	0.0024
12	0.075	0.069	0.07	0.071	0.012	0.0026
13	0.072	0.082	0.069	0.074	0.015	0.0056
14	0.066	0.067	0.064	0.066	0.006	0.0012
15	0.064	0.065	0.067	0.065	0.006	0.0012
16	0.065	0.066	0.066	0.066	0.006	0.0005
17	0.092	0.085	0.096	0.091	0.031	0.0045
19	0.069	0.067	0.070	0.069	0.009	0.0012
20	0.062	0.063	0.064	0.063	0.003	0.0008
23	0.076	0.079	0.079	0.078	0.018	0.0014
25	0.066	0.068	0.067	0.067	0.007	0.0008
26	0.068	0.067	0.068	0.068	0.008	0.0005
27	0.064	0.063	0.064	0.064	0.004	0.0005
28	0.069	0.067	0.067	0.068	0.008	0.0009
29	0.066	0.064	0.066	0.065	0.006	0.0009
30	0.065	0.063	0.064	0.064	0.004	0.0008
31	0.079	0.073	0.077	0.076	0.017	0.0025
32	0.07	0.072	0.074	0.072	0.012	0.0016
33	0.065	0.066	0.066	0.066	0.006	0.0005
34	0.067	0.066	0.065	0.066	0.006	0.0008
37	0.070	0.072	0.072	0.071	0.012	0.0009
38	0.065	0.064	0.067	0.065	0.006	0.0012
42	0.069	0.070	0.072	0.070	0.011	0.0012
NC	0.060	0.060	0.059	0.060	-	0.0005
PC	0.064	0.066	0.064	0.065	0.005	0.0009

Table S3 OD growth values of *A. faecalis*, *P. fulva*, and *P. putida* isolates following incubation at 25°C. NC: Negative Control; PC: Positive Control.

25ºC						
**Isolate**	**Rep 1**	**Rep 2**	**Rep 3**	**Mean OD**	**OD corrected**	**Standard deviation**
1	1.230	1.226	1.235	1.230	1.176	0.0037
2	1.124	1.129	1.167	1.140	1.086	0.0192
3	1.297	1.243	1.376	1.305	1.251	0.0546
4	1.192	1.228	1.192	1.204	1.150	0.0170
5	1.269	1.290	1.285	1.281	1.227	0.0090
6	1.206	1.125	1.119	1.150	1.096	0.0397
7	1.029	1.095	0.967	1.030	0.976	0.0523
9	1.198	1.225	1.278	1.234	1.179	0.0332
10	1.037	1.118	1.123	1.093	1.038	0.0394
11	0.568	0.575	0.538	0.560	0.506	0.0160
12	1.157	1.150	1.104	1.137	1.083	0.0235
13	1.212	1.190	1.160	1.187	1.133	0.0213
14	1.239	1.270	1.302	1.270	1.216	0.0257
15	1.124	1.349	1.281	1.251	1.197	0.0942
16	1.103	1.140	1.091	1.111	1.057	0.0209
17	1.282	1.256	1.314	1.284	1.230	0.0237
19	0.472	0.469	0.474	0.472	0.417	0.0021
20	0.480	0.476	0.487	0.481	0.427	0.0045
23	0.479	0.474	0.554	0.502	0.448	0.0366
25	0.481	0.496	0.481	0.486	0.432	0.0071
26	0.504	0.517	0.523	0.515	0.460	0.0079
27	1.296	1.324	1.413	1.344	1.290	0.0499
28	0.476	0.494	0.530	0.500	0.446	0.0224
29	0.620	0.603	0.649	0.624	0.570	0.0190
30	0.424	0.423	0.420	0.422	0.368	0.0017
31	1.037	1.025	1.021	1.028	0.973	0.0068
32	0.960	1.035	1.038	1.011	0.957	0.0361
33	1.226	1.472	1.029	1.242	1.188	0.1812
34	0.548	0.552	0.553	0.551	0.497	0.0022
37	0.456	0.465	0.474	0.465	0.411	0.0073
38	1.063	1.057	0.723	1.060	1.006	0.0030
42	0.409	0.288	0.363	0.353	0.299	0.0499
NC	0.055	0.055	0.053	0.054	-	0.0010
PC	0.846	0.830	0.863	0.847	0.792	0.0165

Table S4 OD growth values of *A. faecalis*, *P. fulva* and *P. putida* isolates following incubation at 37°C. NC: Negative Control; PC: Positive Control. Values highlighted in red were excluded from the analysis because they were considered outliers.

37ºC						
**Isolate**	**Rep 1**	**Rep 2**	**Rep 3**	**Mean OD**	**OD corrected**	**Standard deviation**
1	1.065	1.075	1.112	1.084	1.029	0.0202
2	1.239	1.289	1.266	1.265	1.209	0.0204
3	1.009	0.907	0.811	0.909	0.854	0.0808
4	1.121	1.075	1.103	1.100	1.044	0.0189
5	0.896	0.691	0.943	0.843	0.788	0.1094
6	1.067	1.246	1.116	1.143	1.088	0.0755
7	1.012	0.944	0.995	0.984	0.928	0.0289
9	0.898	1.049	1.025	0.991	0.935	0.0663
10	0.978	0.956	0.986	0.973	0.918	0.0127
11	0.920	0.998	1.078	0.999	0.943	0.0645
12	0.880	0.947	0.975	0.934	0.879	0.0399
13	0.950	0.945	0.963	0.953	0.897	0.0076
14	0.990	1.041	1.004	1.012	0.956	0.0215
15	0.953	1.142	1.059	1.051	0.996	0.0773
16	1.098	1.026	1.000	1.041	0.986	0.0415
17	1.101	1.103	1.082	1.095	1.040	0.0095
19	0.724	0.872	0.974	0.857	0.801	0.1026
20	0.900	0.984	0.957	0.947	0.892	0.0350
23	0.810	1.038	1.048	1.043	0.988	0.0050
25	1.071	1.045	1.060	1.059	1.003	0.0107
26	1.048	1.088	1.045	1.060	1.005	0.0196
27	1.429	1.285	1.240	1.318	1.263	0.0806
28	0.793	0.802	0.805	0.800	0.745	0.0051
29	0.896	0.857	0.828	0.860	0.805	0.0279
30	0.902	0.949	0.914	0.922	0.866	0.0199
31	1.360	1.387	1.408	1.385	1.330	0.0196
32	1.411	1.354	1.401	1.389	1.333	0.0249
33	1.142	1.080	1.119	1.114	1.058	0.0256
34	1.001	1.023	1.021	1.015	0.960	0.0099
37	0.825	0.826	0.838	0.830	0.774	0.0059
38	0.777	0.644	0.801	0.741	0.685	0.0691
42	1.075	1.137	1.114	1.109	1.053	0.0256
NC	0.056	0.055	0.055	0.055	-	0.0005
PC	1.029	0.981	1.020	1.010	0.955	0.0208

Table S5 OD growth values of *A. faecalis*, *P. fulva,* and *P. putida* isolates following incubation at 42°C. NC: Negative Control; PC: Positive Control.

				42ºC		
**Isolate**	**Rep 1**	**Rep 2**	**Rep 3**	**Mean OD**	**OD corrected**	**Standard deviation**
1	0.070	0.071	0.07	0.070	0.015	0.0005
2	0.064	0.063	0.062	0.063	0.008	0.0008
3	0.070	0.07	0.069	0.070	0.014	0.0005
4	0.074	0.079	0.079	0.077	0.022	0.0024
5	0.070	0.074	0.074	0.073	0.017	0.0019
6	0.079	0.079	0.085	0.081	0.026	0.0028
7	0.079	0.08	0.083	0.081	0.025	0.0017
9	0.084	0.09	0.098	0.091	0.035	0.0057
10	0.074	0.074	0.074	0.074	0.019	0.0000
11	0.087	0.09	0.092	0.090	0.034	0.0021
12	0.100	0.099	0.107	0.102	0.047	0.0036
13	0.119	0.14	0.147	0.135	0.080	0.0119
14	0.074	0.082	0.076	0.077	0.022	0.0034
15	0.074	0.076	0.082	0.077	0.022	0.0034
16	0.079	0.09	0.098	0.089	0.034	0.0078
17	0.118	0.126	0.131	0.125	0.070	0.0054
19	0.083	0.085	0.084	0.084	0.029	0.0008
20	0.089	0.092	0.097	0.093	0.037	0.0033
23	0.133	0.140	0.149	0.141	0.085	0.0065
25	0.088	0.087	0.086	0.087	0.032	0.0008
26	0.088	0.091	0.095	0.091	0.036	0.0029
27	0.090	0.104	0.107	0.100	0.045	0.0074
28	0.116	0.126	0.129	0.124	0.068	0.0056
29	0.089	0.090	0.088	0.089	0.034	0.0008
30	0.080	0.082	0.085	0.082	0.027	0.0021
31	0.437	0.450	0.494	0.460	0.405	0.0244
32	0.569	0.543	0.536	0.549	0.494	0.0142
33	0.064	0.065	0.066	0.065	0.010	0.0008
34	0.077	0.081	0.085	0.081	0.026	0.0033
37	0.097	0.100	0.101	0.099	0.044	0.0017
38	0.080	0.08	0.073	0.078	0.022	0.0033
42	0.125	0.127	0.122	0.125	0.069	0.0021
NC	0.056	0.057	0.053	0.055	-	0.0017
PC	0.380	0.357	0.371	0.369	0.314	0.0095

Table S6 Corrected OD values, standard deviations (SD), and relative growth (%) of *A. faecalis*, *P. fulva*, and *P. putida* isolates grown in NaCl-free medium (0% NaCl). The 0% NaCl condition was used as the control and was assigned a relative growth value of 100%. NC: Negative Control; PC: Positive Control. Values highlighted in red were excluded from the analysis because they were considered outliers.

				0%			
**Isolate**	**Rep 1**	**Rep 2**	**Rep 3**	**Mean OD**	**OD corrected**	**Standard deviation**	**Relative Growth (%)**
1	1.065	1.075	1.112	1.084	1.029	0.0202	100
2	1.239	1.289	1.266	1.265	1.210	0.0204	100
3	1.009	0.907	0.811	0.909	0.854	0.0808	100
4	1.121	1.075	1.103	1.100	1.045	0.0189	100
5	0.896	0.691	0.943	0.920	0.8642	0.0235	100
6	1.067	1.246	1.116	1.143	1.088	0.0755	100
7	1.012	0.944	0.995	0.984	0.929	0.0289	100
9	0.898	1.049	1.025	0.991	0.936	0.0663	100
10	0.978	0.956	0.986	0.973	0.918	0.0127	100
11	0.920	0.998	1.078	0.999	0.944	0.0645	100
12	0.880	0.947	0.975	0.934	0.879	0.0399	100
13	0.950	0.945	0.963	0.953	0.898	0.0076	100
14	0.990	1.041	1.004	1.012	0.957	0.0215	100
15	0.953	1.142	1.059	1.051	0.996	0.0773	100
16	1.098	1.026	1.000	1.041	0.986	0.0415	100
17	1.101	1.103	1.082	1.095	1.040	0.0095	100
19	0.724	0.872	0.974	0.857	0.802	0.1026	100
20	0.900	0.984	0.957	0.947	0.892	0.0350	100
23	0.810	1.038	1.048	1.043	0.9877	0.0050	100
25	1.071	1.045	1.060	1.059	1.004	0.0107	100
26	1.048	1.088	1.045	1.060	1.005	0.0196	100
27	1.429	1.285	1.240	1.318	1.263	0.0806	100
28	0.793	0.802	0.805	0.800	0.745	0.0051	100
29	0.896	0.857	0.828	0.860	0.805	0.0279	100
30	0.902	0.949	0.914	0.922	0.867	0.0199	100
31	1.360	1.387	1.408	1.385	1.330	0.0196	100
32	1.411	1.354	1.401	1.389	1.334	0.0249	100
33	1.142	1.080	1.119	1.114	1.059	0.0256	100
34	1.001	1.023	1.021	1.015	0.960	0.0099	100
37	0.825	0.826	0.838	0.830	0.775	0.0059	100
38	0.777	0.644	0.801	0.741	0.686	0.0691	100
42	1.075	1.137	1.114	1.109	1.054	0.0256	100
NC	0.056	0.055	0.055	0.055	-	0.0005	100
PC	1.029	0.981	1.020	1.010	0.955	0.0208	100

Table S7 Corrected OD values, SD and relative growth (%) of *A. faecalis*, *P. fulva*, and *P. putida* isolates grown in medium supplemented with 1% NaCl. Relative growth was calculated with

respect to the 0% NaCl control. NC: Negative Control; PC: Positive Control. Values highlighted in red were excluded from the analysis because they were considered outliers.

				1%			
**Isolate**	**Rep 1**	**Rep 2**	**Rep 3**	**Mean OD**	**OD corrected**	**Standard deviation**	**Relative Growth (%)**
1	0.754	0.621	0.593	0.656	0.599	0.0702	58.24
2	0.486	0.477	0.571	0.511	0.455	0.0424	37.57
3	0.506	0.537	0.512	0.518	0.462	0.0134	54.06
4	0.711	0.684	0.697	0.697	0.641	0.0110	61.30
5	0.813	0.694	0.65	0.719	0.662	0.0689	76.64
6	0.576	0.567	0.517	0.553	0.497	0.0260	45.65
7	0.581	0.482	0.577	0.547	0.490	0.0458	52.74
9	0.507	0.612	0.551	0.557	0.500	0.0431	53.42
10	0.592	0.62	0.712	0.641	0.585	0.0513	63.69
11	0.975	1.01	0.998	0.994	0.938	0.0145	99.33
12	0.659	0.615	0.604	0.626	0.569	0.0238	64.77
13	0.703	0.691	0.667	0.687	0.630	0.0150	70.19
14	0.897	0.675	0.646	0.661	0.604	0.0145	63.09
15	0.815	1.009	0.971	0.932	0.875	0.0839	87.85
16	0.561	0.495	0.57	0.542	0.485	0.0334	49.22
17	0.622	0.667	0.647	0.645	0.589	0.0184	56.60
19	0.656	0.869	0.941	0.905	0.848	0.0360	92.93
20	1.002	0.997	0.968	0.989	0.932	0.0150	93.64
23	0.925	1.003	0.995	0.974	0.918	0.0350	94.26
25	0.612	0.861	0.96	0.911	0.854	0.0495	94.35
26	0.914	0.954	0.951	0.940	0.883	0.0182	94.36
27	0.71	0.661	0.682	0.684	0.628	0.0201	95.51
28	0.979	1.025	1.009	1.004	0.948	0.0191	92.39
29	0.533	0.885	0.88	0.766	0.709	0.1648	92.96
30	0.927	0.918	0.931	0.925	0.869	0.0054	93.46
31	1.052	1.01	1.065	1.042	0.986	0.0235	95.74
32	1.082	1.267	1.175	1.175	1.118	0.0755	95.75
33	0.213	0.476	0.467	0.472	0.415	0.0045	94.65
34	0.891	0.886	0.937	0.905	0.848	0.0230	94.09
37	0.802	0.798	0.828	0.809	0.753	0.0133	92.68
38	0.314	0.317	0.292	0.308	0.251	0.0111	91.73
42	0.168	0.214	0.247	0.210	0.153	0.0324	94.62
NC	0.057	0.056	0.057	0.057	-	0.0005	-
PC	1.201	1.152	1.163	1.172	1.115	0.0210	116.75

Table S8 Corrected OD values, SD, and relative growth (%) of *A. faecalis*, *P. fulva*, and *P. putida* isolates grown in medium supplemented with 2% NaCl. Relative growth was calculated with respect to the 0% NaCl

control. NC: Negative Control; PC: Positive Control. Values highlighted in red were excluded from the analysis because they were considered outliers.

					2%		
**Isolate**	**Rep 1**	**Rep 2**	**Rep 3**	**Mean OD**	**OD corrected**	**Standard deviation**	**Relative Growth (%)**
1	0.734	0.735	0.732	0.734	0.678	0.0012	65.92
2	0.71	0.607	0.611	0.643	0.587	0.0476	48.54
3	0.523	0.61	0.638	0.590	0.535	0.0490	62.65
4	0.66	0.831	0.783	0.758	0.703	0.0720	67.24
5	0.741	0.747	0.77	0.753	0.697	0.0125	80.70
6	0.691	0.654	0.616	0.654	0.598	0.0306	55.00
7	0.531	0.56	0.566	0.552	0.497	0.0153	53.50
9	0.627	0.675	0.659	0.654	0.598	0.0200	63.93
10	0.684	0.545	0.655	0.628	0.573	0.0599	62.39
11	0.833	0.934	0.926	0.898	0.842	0.0458	89.23
12	1.27	1.309	1.326	1.302	1.246	0.0234	141.79
13	1.359	1.407	1.341	1.369	1.314	0.0279	146.29
14	1.236	1.283	1.179	1.233	1.177	0.0425	123.03
15	0.563	0.487	0.777	0.525	0.470	0.0380	47.16
16	0.487	0.463	0.494	0.481	0.426	0.0133	43.21
17	0.606	0.568	0.642	0.605	0.550	0.0302	52.89
19	0.933	0.969	0.905	0.936	0.880	0.0262	109.77
20	0.893	0.902	0.912	0.902	0.847	0.0078	94.96
23	0.949	1.019	0.968	0.979	0.923	0.0296	93.49
25	1.142	1.079	1.048	1.090	1.034	0.0391	103.02
26	1.041	1.075	0.998	1.038	0.983	0.0315	97.78
27	0.71	0.659	0.596	0.655	0.600	0.0466	47.48
28	1.005	0.995	1.031	1.010	0.955	0.0152	128.19
29	1.053	1.033	1.046	1.044	0.989	0.0083	122.82
30	1.001	1.008	0.96	0.990	0.934	0.0212	107.77
31	0.848	0.748	0.837	0.811	0.756	0.0448	56.82
32	0.941	0.897	0.91	0.916	0.861	0.0185	64.52
33	1.069	1.01	1.063	1.047	0.992	0.0265	93.68
34	1.01	0.957	0.965	0.977	0.922	0.0233	96.05
37	0.805	0.799	0.799	0.801	0.746	0.0028	96.22
38	0.705	0.907	0.7	0.771	0.715	0.0964	104.28
42	0.863	0.772	0.674	0.770	0.714	0.0772	67.78
NC	0.056	0.055	0.055	0.055	-	0.0005	-
PC	1.304	1.451	1.448	1.401	1.346	0.0686	140.94

Table S9 Corrected OD values, SD, and relative growth (%) of *A. faecalis*, *P. fulva* and *P. putida* isolates grown in medium supplemented with 4% NaCl. Relative growth was calculated with respect to the 0% NaCl

control. NC: Negative Control; PC: Positive Control. Values highlighted in red were excluded from the analysis because they were considered outliers.

				4%			
**Isolate**	**Rep 1**	**Rep 2**	**Rep 3**	**Mean OD**	**OD corrected**	**Standard deviation**	**Relative Growth (%)**
1	0.79	0.861	0.863	0.838	0.779	0.0340	0.779
2	0.814	0.843	0.817	0.825	0.766	0.0130	0.766
3	0.828	0.607	0.555	0.581	0.522	0.0260	0.522
4	0.603	0.495	0.468	0.522	0.463	0.0583	0.463
5	0.594	0.614	0.646	0.618	0.559	0.0214	0.559
6	0.572	0.597	0.503	0.557	0.499	0.0398	0.499
7	0.77	0.434	0.441	0.438	0.379	0.0035	0.379
9	0.764	0.531	0.517	0.524	0.465	0.0070	0.465
10	0.495	0.554	0.643	0.564	0.505	0.0608	0.505
11	0.62	0.662	0.586	0.623	0.564	0.0311	0.564
12	0.388	0.37	0.521	0.426	0.368	0.0673	0.368
13	0.522	0.413	0.37	0.435	0.376	0.0640	0.376
14	0.63	0.614	0.553	0.599	0.540	0.0332	0.540
15	0.834	0.546	0.587	0.567	0.508	0.0205	0.508
16	0.406	0.398	0.49	0.431	0.373	0.0416	0.373
17	0.485	0.754	0.532	0.509	0.450	0.0235	0.450
19	0.8	0.702	0.802	0.768	0.709	0.0467	0.709
20	0.707	0.686	0.781	0.725	0.666	0.0407	0.666
23	0.931	0.763	0.701	0.798	0.740	0.0972	0.740
25	1.245	1.188	1.256	1.230	1.171	0.0298	1.171
26	0.751	0.722	0.745	0.739	0.681	0.0125	0.681
27	0.858	0.804	0.826	0.829	0.771	0.0222	0.771
28	0.679	0.842	0.841	0.787	0.729	0.0766	0.729
29	0.847	0.764	0.854	0.822	0.763	0.0409	0.763
30	0.779	0.726	0.605	0.703	0.645	0.0728	0.645
31	0.869	0.704	0.57	0.787	0.728	0.0825	0.728
32	0.649	0.676	0.734	0.686	0.628	0.0355	0.628
33	1.001	0.994	1.012	1.002	0.944	0.0074	0.944
34	0.671	0.716	0.726	0.704	0.646	0.0239	0.646
37	0.567	0.572	0.531	0.557	0.498	0.0183	0.498
38	0.286	0.392	0.334	0.337	0.279	0.0433	0.279
42	0.224	0.239	0.249	0.237	0.179	0.0103	0.179
NC	0.062	0.057	0.057	0.059	-	0.0024	-
PC	0.524	0.568	0.723	0.605	0.546	0.0854	57.17

Table S10 Corrected OD values, SD, and relative growth (%) of *A. faecalis*, *P. fulva* and *P. putida* isolates grown in medium supplemented with 6% NaCl. Relative growth was calculated with respect to the 0% NaCl

control. NC: Negative Control; PC: Positive Control. Values highlighted in red were excluded from the analysis because they were considered outliers.

					6%		
**Isolate**	**Rep 1**	**Rep 2**	**Rep 3**	**Mean OD**	**OD corrected**	**Standard deviation**	**Relative Growth (%)**
1	0.146	0.156	0.164	0.155	0.095	0.0074	0.095
2	0.204	0.266	0.277	0.249	0.189	0.0321	0.189
3	0.166	0.215	0.195	0.192	0.132	0.0201	0.132
4	0.16	0.158	0.138	0.152	0.092	0.0099	0.092
5	0.111	0.121	0.117	0.116	0.056	0.0041	0.056
6	0.136	0.144	0.179	0.153	0.093	0.0187	0.093
7	0.151	0.171	0.145	0.156	0.095	0.0111	0.095
9	0.135	0.144	0.176	0.152	0.091	0.0176	0.091
10	0.12	0.119	0.135	0.125	0.064	0.0073	0.064
11	0.087	0.188	0.194	0.156	0.096	0.0491	0.096
12	0.101	0.105	0.107	0.104	0.044	0.0025	0.044
13	0.124	0.119	0.1	0.114	0.054	0.0103	0.054
14	0.125	0.118	0.123	0.122	0.062	0.0029	0.062
15	0.2	0.223	0.59	0.212	0.151	0.0115	0.151
16	0.151	0.131	0.186	0.156	0.096	0.0227	0.096
17	0.227	0.188	0.212	0.209	0.149	0.0161	0.149
19	0.257	0.236	0.22	0.238	0.177	0.0152	0.177
20	0.227	0.242	0.213	0.227	0.167	0.0118	0.167
23	0.728	0.538	0.429	0.484	0.423	0.0545	0.423
25	0.684	0.975	0.823	0.899	0.839	0.0760	0.839
26	0.254	0.309	0.296	0.286	0.226	0.0235	0.226
27	0.164	0.173	0.14	0.159	0.099	0.0139	0.099
28	0.286	0.228	0.226	0.247	0.186	0.0278	0.186
29	0.343	0.31	0.328	0.327	0.267	0.0135	0.267
30	0.241	0.269	0.211	0.240	0.180	0.0237	0.180
31	0.434	0.643	0.55	0.542	0.482	0.0855	0.482
32	0.632	0.602	0.622	0.619	0.558	0.0125	0.558
33	0.321	0.344	0.339	0.335	0.274	0.0099	0.274
34	0.243	0.248	0.176	0.222	0.162	0.0328	0.162
37	0.192	0.259	0.217	0.223	0.162	0.0276	0.162
38	0.068	0.071	0.072	0.070	0.010	0.0017	0.010
42	0.104	0.112	0.106	0.107	0.047	0.0034	0.047
NC	0.06	0.059	0.062	0.060	-	0.0012	-
PC	0.074	0.196	0.246	0.172	0.112	0.0722	11.728

Table S11 Corrected OD values, SD, and relative growth (%) of *A. faecalis*, *P. fulva* and *P. putida* isolates grown in medium supplemented with 8% NaCl. Relative growth was calculated with respect to the 0% NaCl control. NC: Negative Control; PC: Positive Control.

8%

Isolate	Rep 1	Rep 2	Rep 3	Mean OD	OD corrected	Standard deviation	Relative Growth (%)
1	0.066	0.067	0.066	0.066	0.008	0.0005	0.008
2	0.085	0.077	0.075	0.079	0.021	0.0043	0.021
3	0.073	0.087	0.119	0.093	0.035	0.0193	0.035
4	0.073	0.068	0.066	0.069	0.011	0.0029	0.011
5	0.065	0.064	0.066	0.065	0.007	0.0008	0.007
6	0.068	0.074	0.072	0.071	0.013	0.0025	0.013
7	0.067	0.066	0.068	0.067	0.009	0.0008	0.009
9	0.071	0.071	0.068	0.070	0.012	0.0014	0.012
10	0.067	0.074	0.069	0.070	0.012	0.0029	0.012
11	0.061	0.067	0.063	0.064	0.006	0.0025	0.006
12	0.063	0.063	0.066	0.064	0.006	0.0014	0.006
13	0.066	0.067	0.068	0.067	0.009	0.0008	0.009
14	0.066	0.068	0.064	0.066	0.008	0.0016	0.008
15	0.062	0.064	0.064	0.063	0.005	0.0009	0.005
16	0.062	0.064	0.067	0.064	0.006	0.0021	0.006
17	0.063	0.065	0.062	0.063	0.005	0.0012	0.005
19	0.059	0.063	0.063	0.062	0.004	0.0019	0.004
20	0.066	0.069	0.196	0.110	0.052	0.0606	0.052
23	0.426	0.465	0.416	0.436	0.378	0.0211	0.378
25	0.703	0.727	0.792	0.741	0.683	0.0376	0.683
26	0.071	0.071	0.066	0.069	0.011	0.0024	0.011
27	0.067	0.068	0.064	0.066	0.008	0.0017	0.008
28	0.066	0.064	0.065	0.065	0.007	0.0008	0.007
29	0.064	0.07	0.077	0.070	0.012	0.0053	0.012
30	0.064	0.063	0.063	0.063	0.005	0.0005	0.005
31	0.145	0.12	0.114	0.126	0.068	0.0134	0.068
32	0.134	0.147	0.176	0.152	0.094	0.0176	0.094
33	0.066	0.067	0.064	0.066	0.008	0.0012	0.008
34	0.06	0.061	0.06	0.060	0.002	0.0005	0.002
37	0.079	0.065	0.074	0.073	0.015	0.0058	0.015
38	0.07	0.065	0.095	0.077	0.019	0.0131	0.019
42	0.073	0.074	0.074	0.074	0.016	0.0005	0.016
NC	0.058	0.057	0.059	0.058	-	0.0008	-
PC	0.063	0.063	0.065	0.064	0.006	0.0009	0.628

Table S12 Viable bacterial counts (log_{10} CFU/mL) of *P. fulva, P. putida,* and *A. faecalis* isolates at each sampling time during the nutrient starvation assay.

	log_{10} (CFU/mL)							
**Isolate**	**0 hours**	**24 hours**	**48 hours**	**72 hours**	**7 days**	**14 days**	**4 weeks**	**6 weeks**
1	7.77	>8.78	TNTC	>8.78	>8.78	>8.78	8.22	7.46

2	>8.78	>8.78	TNTC	>8.78	>8.78	>8.78	8.39	7.47
3	7.62	>8.78	TNTC	>8.78	>8.78	>8.78	8.24	7.53
4	7.83	>8.78	TNTC	>8.78	>8.78	>8.78	8.07	7.35
5	7.52	>8.78	TNTC	>8.78	>8.78	>8.78	8.19	7.36
6	7.58	>8.78	TNTC	>8.78	>8.78	>8.78	8.07	7.43
7	7.55	>8.78	TNTC	>8.78	>8.78	>8.78	7.89	7.43
9	7.74	>8.78	TNTC	>8.78	>8.78	>8.78	8.21	7.46
10	7.63	>8.78	TNTC	>8.78	>8.78	>8.78	8.19	7.46
11	7.03	>8.78	TNTC	>8.78	>8.78	>8.78	8.20	7.45
12	7.54	>8.78	TNTC	>8.78	>8.78	>8.78	8.22	7.39
13	7.45	>8.78	TNTC	>8.78	>8.78	>8.78	8.32	7.66
14	7.51	>8.78	TNTC	>8.78	>8.78	>8.78	7.97	7.45
15	7.42	>8.78	TNTC	>8.78	>8.78	>8.78	8.05	7.38
16	>8.78	8.15	TNTC	>8.78	>8.78	>8.78	8.42	7.44
17	>8.78	>8.78	TNTC	>8.78	>8.78	>8.78	8.07	7.51
19	>8.78	>8.78	TNTC	>8.78	>8.78	>8.78	8.34	7.26
20	>8.78	>8.78	TNTC	>8.78	>8.78	>8.78	8.28	7.32
23	>8.78	>8.78	TNTC	>8.78	>8.78	>8.78	8.28	7.53
25	>8.78	>8.78	TNTC	>8.78	>8.78	>8.78	8.58	7.42
26	>8.78	>8.78	TNTC	>8.78	>8.78	>8.78	8.12	7.52
27	>8.78	>8.78	TNTC	>8.78	>8.78	>8.78	8.18	7.40
28	>8.78	>8.78	TNTC	>8.78	>8.78	>8.78	8.23	7.43
29	>8.78	>8.78	TNTC	>8.78	>8.78	>8.78	8.10	7.44
30	>8.78	>8.78	TNTC	>8.78	>8.78	>8.78	8.25	7.47
31	>8.78	>8.78	TNTC	>8.78	>8.78	>8.78	8.24	7.37
32	>8.78	>8.78	TNTC	>8.78	>8.78	>8.78	8.12	7.42
33	7.61	>8.78	TNTC	>8.78	>8.78	>8.78	7.79	7.56
34	>8.78	>8.78	TNTC	>8.78	>8.78	>8.78	8.56	7.37
37	>8.78	8.24	TNTC	>8.78	>8.78	>8.78	8.12	7.48
38	>8.78	>8.78	TNTC	>8.78	>8.78	>8.78	8.68	7.41
42	7.59	>8.78	TNTC	>8.78	>8.78	>8.78	8.15	7.55
NC	0.00	0.00	0.00	0.00	0.00	0.00	0.00	0.00
PC	7.26	>8.78	>8.78	>8.78	>8.78	>8.78	8.26	7.35

Table S13 OD values obtained for each isolate in eight replicates, together with the mean OD, and the corresponding standard deviation, used for biofilm formation analysis.

	**Isolate 1**	**Isolate 2**	**Isolate 3**	**Isolate 4**	**Isolate 5**	**Isolate 6**	**Isolate 7**	**Isolate 9**	**Isolate 10**
Rep 1	0.070	0.051	0.061	0.067	0.068	0.075	0.072	0.084	0.068
Rep 2	0.107	0.048	0.059	0.079	0.068	0.082	0.058	0.068	0.072
Rep 3	0.123	0.066	0.089	0.077	0.082	0.068	0.062	0.066	0.076

Rep 4	0.111	0.061	0.075	0.059	0.057	0.064	0.054	0.072	0.079
Rep 5	0.118	0.053	0.050	0.060	0.073	0.052	0.059	0.064	0.068
Rep 6	0.092	0.056	0.098	0.062	0.075	0.080	0.066	0.052	0.095
Rep 7	0.118	0.054	0.087	0.102	0.084	0.070	0.060	0.081	0.076
Rep 8	0.082	0.053	0.055	0.065	0.059	0.057	0.072	0.084	0.122
**Mean**	0.103	0.055	0.072	0.071	0.071	0.069	0.063	0.071	0.076
**SD**	0.018	0.005	0.017	0.013	0.009	0.010	0.006	0.010	0.009

	**Isolate 11**	**Isolate 12**	**Isolate 13**	**Isolate 14**	**Isolate 15**	**Isolate 16**	**Isolate 17**	**Isolate 19**	**Isolate 20**
Rep 1	0.377	0.149	0.097	0.094	0.073	0.081	0.061	0.277	0.203
Rep 2	0.232	0.130	0.092	0.091	0.131	0.054	0.072	0.243	0.201
Rep 3	0.234	0.148	0.111	0.091	0.097	0.057	0.091	0.164	0.185
Rep 4	0.252	0.129	0.090	0.086	0.137	0.051	0.058	0.173	0.225
Rep 5	0.246	0.157	0.087	0.081	0.121	0.048	0.155	0.187	0.184
Rep 6	0.299	0.201	0.073	0.098	0.141	0.055	0.145	0.199	0.173
Rep 7	0.208	0.120	0.085	0.086	0.084	0.067	0.139	0.206	0.183
Rep 8	0.224	0.181	0.109	0.099	0.086	0.052	0.072	0.173	0.211
**Mean**	0.242	0.152	0.093	0.091	0.109	0.058	0.099	0.203	0.196
**SD**	0.027	0.026	0.012	0.006	0.025	0.010	0.038	0.037	0.016

	**Isolate 23**	**Isolate 25**	**Isolate 26**	**Isolate 27**	**Isolate 28**	**Isolate 29**	**Isolate 30**	**Isolate 31**	**Isolate 32**
Rep 1	0.184	0.26	0.396	0.055	0.183	0.396	0.254	0.309	0.245
Rep 2	0.195	0.223	0.372	0.057	0.132	0.481	0.291	0.306	0.311
Rep 3	0.204	0.217	0.296	0.059	0.177	0.471	0.225	0.332	0.248
Rep 4	0.151	0.245	0.327	0.061	0.166	0.422	0.228	0.259	0.214
Rep 5	0.195	0.174	0.24	0.054	0.158	0.465	0.230	0.384	0.456
Rep 6	0.122	0.194	0.265	0.056	0.152	0.425	0.251	0.328	0.278
Rep 7	0.146	0.234	0.289	0.075	0.158	0.346	0.219	0.229	0.249
Rep 8	0.155	0.233	0.372	0.069	0.223	0.377	0.213	0.335	0.272
**Mean**	0.169	0.223	0.320	0.061	0.169	0.423	0.239	0.310	0.260
**SD**	0.028	0.026	0.053	0.007	0.025	0.045	0.024	0.045	0.028

	**Isolate 33**	**Isolate 34**	**Isolate 37**	**Isolate 38**	**Isolate 42**	**NC**	**PC**
Rep 1	0.075	0.271	0.278	0.126	0.051	0.050	0.241
Rep 2	0.142	0.259	0.257	0.148	0.05	0.048	0.297
Rep 3	0.068	0.307	0.199	0.169	0.059	0.052	0.372
Rep 4	0.072	0.26	0.339	0.149	0.056	0.051	0.350
Rep 5	0.067	0.201	0.23	0.159	0.136	0.053	0.306
Rep 6	0.074	0.206	0.288	0.119	0.053	0.046	0.388
Rep 7	0.08	0.221	0.18	0.13	0.053	0.046	0.399
Rep 8	0.062	0.248	0.213	0.107	0.049	0.044	0.428
**Mean**	0.071	0.247	0.248	0.138	0.053	0.049	0.348
**SD**	0.006	0.033	0.049	0.020	0.003	0.003	0.058
